# Curious and Fatal Case of Mania, by Dr. Brierre

**Published:** 1826-07

**Authors:** 


### CURIOUS AND FATAL CASE OF MANIA.*

The pathology of mental alienation is far from having arrived at perfection. Many observations are yet wanting to elucidate its etiology also. On this account we present the following case with its dissection. Mad. J. arrived at her 42d year, without evincing any symptom of mental alienation. She became married at the age of 34, but was soon after obliged to separate from her husband. For eight years more she continued to enjoy good health and spirits. During the three months preceding the present report, her friends were surprised to find her become extremely voluble, talking incessantly for hours together, but with intervals of quietude. These intervals became shorter and shorter, and she was sent into the Maison de Santé of St. Colombe. Our author saw the patient on the 15th May, 1825. Her face was rather flushed —and her eyes rather wild. Her conversation was incoherent. In the evening, after a very joyous fit, she had a paroxysm of hysteria. 18th. Her loquacity being great—her face flushed—with other symptoms of considerable irritation, she was bled. 20th, Bled again; and from

---

* Dr. Brierre. Bullet. de L'Athenée de Médecine.

great agitation she passed into a state of comparative tranquillity—and some rationality. *2d June.* The insanity now presented a different character. From obstinate taciturnity, she would burst out into loud laughter or declamation. Her tongue was white; but she had no febrile symptoms. Her apartment was darkened, and she was put upon low diet. She had occasional attacks of hysteria. During the whole of the 6th June, she appeared in a profound sleep—her muscles contracted—her jaws clenched—her arms were cataleptic. 7th, Continues in the same state—her face flushed—body covered with sweat—pulse full and frequent. She took no nourishment during the last two days. In the evening she rallied, and spoke more rationally. Her skin was turning of a dull yellow colour. 8th and 9th. Relapse into the same lethargic state, with cataleptic condition of the upper and lower extremities. 11th, The catalepsy was well marked in the limbs, which preserved whatever attitude they were placed in. Suspecting that she was feigning, they placed her on her feet, and she soon fell to the ground. 12th and 13th, In the same state. 15th, She emaciates rapidly. Her gums are pale—her feet cold—she broke silence to day, and complained of weight at the epigastrium. Twelve leeches were applied there. 19th, During the last three days the patient spoke not, and took no aliment. All at once she roused herself—her eyes became furious, and her looks haggard—she tried to bite those around her—pulse small and feeble. On the 8th July, we find her affected with sickness and vomiting, which ceased on the 11th. At this time she had been 12 days without a stool, in spite of strong glysters, &c. From this period till the 14th, (the day of her death) she got worse and worse.

*Dissection.* With the exception of the uterus, there was nothing particular in the thoracic, abdominal, or pelvic viscera. The internal surface of the uterus was red, and covered with a puriform secretion. The anterior portions of the two anterior lobes of the brain were softened, and of a pale yellow colour. This alteration was about a line in depth. There was no serum in the ventricles. The medullary substance was very hard, as was also the medulla oblongata. The right thalamus nervi optici presented a softened portion half an inch in extent, which readily separated from the sound substance underneath. The membranes of the brain were sound, and there was a small quantity of serous fluid between them.

The cause of the mental alienation, our author thinks, must be sought in the condition of the uterus. Its internal surface had been evidently, for some time, in a state of inflammation. The brain, he imagines, became affected, sympathetically, from the uterus. The phenomena of hysteria and catalepsy, will, of course, be readily placed to the account of the uterine affection.

